# Pro inflammatory interleukins and thyroid function in Naswar (dipping tobacco) users: a case control study

**DOI:** 10.1186/s12902-016-0127-5

**Published:** 2016-08-11

**Authors:** Faiza Sajid, Samina Bano

**Affiliations:** Clinical Biochemistry and Psychopharmacology Research Unit, Department of Biochemistry, University of Karachi, Karachi, 75270 Pakistan

**Keywords:** Smokeless tobacco, Naswar, Interleukins, Free triiodothyronine, Free thyroxine, Thyroid stimulating hormone

## Abstract

**Background:**

Naswar is a type of finely ground, moistened smokeless dipping tobacco product being commonly used in Pakistan. Although, nicotine is the most important psychoactive agent present in Naswar, it also exerts immunosuppressive effects and could alter the levels of cytokines. Additionally, the effects of Naswar consumption on thyroid hormones are not known.

**Methods:**

Eighty healthy males aged 16–43 years were selected for the study and were divided into a control group comprising 31 healthy subjects with no history of tobacco use in any form, with age matched test group comprising 49 exclusive Naswar users who were consuming Naswar for at least 1 year. Estimation of serum interleukin (IL)-1β, IL-6, free thyroxine (FT4), free triiodothyronine (FT3) and thyroid stimulating hormone (TSH) was carried out. The data was analyzed by statistical programme (SPSS) using student's independent samples *t*-test. One way Anova followed by post hoc Tukey test was applied to assess parameters in Naswar users grouped according to duration of Naswar usage. Pearson’s correlation coefficient was applied to assess correlations between parameters.

**Results:**

IL-1β was found to be significantly lowered in Naswar users compared to the control group whereas serum FT3 and FT4 levels in Naswar users were significantly raised compared to the control group. However, no differences in the levels of serum IL-6 and TSH between Naswar users and the control group were found. Also, serum FT3 and FT4 were consistently raised whereas IL-1β was lowered in Naswar users irrespective of duration of Naswar consumption. IL-1β was negatively correlated with FT3 in Naswar users.

**Conclusion:**

The findings suggest that Naswar users might be in an immune suppressive state as evident by the lowered levels of interleukin 1β. Additionally, alterations in the levels of thyroid hormones signify the impact of Naswar consumption on thyroid function.

## Background

Naswar (sometimes also called niswar) is a dipping smokeless tobacco product commonly used in Pakistan, India, Iran, Afghanistan and in South Africa [1]. In Pakistan, it is widely used by the Pushton ethnic group where it constitutes an integral part of their cultural heritage being considered completely harmless. The sun-dried tobacco leaves, belonging to Nicotina rustica species, are finely ground and then mixed with calcium oxide and wood ash. Flavorings such as cardamom, menthol and coloring agents such as indigo are also added. These ingredients are placed in a cement-lined cavity and pounded with a weighty wooden hammer. After addition of water, the material is rolled into balls. Packed in small polythene bags, the size of tea bags, a pinch of Naswar (about 1 g) is placed between cheek and gum several times during the course of the day for about 30 min before being spat out [2].

Naswar has been implicated in the causation of oral cavity and esophageal cancers [3]. It contains increased concentrations of oncogenic substances such as nitrosamines and various toxic metals including cadmium, arsenic, lead and chromium [2]. Exposure to smokeless tobacco (ST) causes systemic absorption of various toxic metals [4] that might cause altered levels of cytokines. Cytokines are low-molecular-weight proteins that are secreted by lymphocytes and macrophages [5] and could be broadly classified into two major types namely the pro inflammatory and the anti-inflammatory cytokines. The pro inflammatory cytokines as the name suggests are involved in inflammatory processes for eg Interleukin (IL)-1, IL-6, interferon (IFN)- γ and tumor necrosis factor(TNF)-α whereas the anti-inflammatory interleukins for e.g., IL-13, IL-10 and IL-4, inhibit the immune response and also decrease the production of pro inflammatory cytokines. Cadmium chloride at low concentrations causes increased concentrations of mRNA forIL-1α, IL-1β, IL-8, TNFα as well as for IL-6 but at higher concentrations, it causes depression of the accumulation of mRNA for IL-1α, IL-1β and TNFα but not for IL-6 and IL-8 [6].

Tobacco smoking exerts an inflammatory effect on alveolar macrophages leading to the production of proinflammatory cytokines such as IL-6 and IL-1β [7, 8]. However, although the harmful effects of tobacco smoke are avoided in smokeless tobacco products, the presence of nicotine in Naswar [2] could cause alterations in the levels of cytokines as nicotine has been to shown to exert immunosuppressive effects [9]. The nicotinic acetylcholine receptor-α7 (α7nAChR) subunit inhibits the synthesis of pro inflammatory cytokine by cholinergic anti- inflammatory pathway, the latter being involved in immune response [10]. A study that evaluated systemic immune markers involved in inflammation and immunity showed decreased levels of seven markers of inflammation including proinflammatory (IL-1β) and anti-inflammatory cytokines (IL-1Ra) in current smokers compared to non-smokers [11]. Additionally several other studies have also shown the association of cigarette smoking with increased levels of C-reactive protein and decreased levels of IL-1β in vitro and in animal models [12–15] thus emphasizing immuno-supppressive effects of cigarette smoking on the cytokines and in turn pointing towards immune-suppressive effects of nicotine [16]. However, the effect of Naswar consumption on systemic proinflammatory interleukins is not known.

Several studies indicate that smokers exhibit a decreased risk of developing thyroid peroxidase and thyroglobulin antibodies and are protected against autoimmune hypothyroidism [17–20]. Acting via α7-nicotinic-acetylcholine receptor (nAChR), nicotine increases the T-mediated immune suppression of lymphocytes. Nicotine has also been shown to alter the autoimmune profile from pathogenic Th 1 and Th17 responses to protective Th 2 response. On the other hand, smokers have also been shown to be at an increased risk of developing Graves ophthalmopathy [21] and that in turn might be mediated by Th 2 responses [22]. Additionally, the presence of various toxins such as thiocyanate could also be one of the causes of development of graves ophthalmopathy in cigarette smokers [23]. However, the effects of smokeless tobacco consumption on thyroid hormone levels are not known.

With this background, the present study was designed to evaluate the circulatory levels of proinflammatory markers in relation to Naswar consumption. Additionally, the levels of thyroid hormones were also determined in order to ascertain the endocrine effect of nicotine in the smokeless tobacco. Additionally, as proinflammatory cytokines have been shown to influence thyroid function [24] any association between IL-1β and IL-6 levels versus FT3, FT4 and TSH in Naswar users was also evaluated.

## Methods

### Study Design

Eighty healthy male subjects aged 16–43 years were recruited for this case control study, following written, informed consent. Females were excluded as Naswar is predominantly used by the male pushtoon ethnic group and is rarely used by the females in Pakistan.

Subjects were divided into a control group comprising 31 healthy subjects with no history of tobacco use in any form, with age matched test group comprising 49 exclusive Naswar users who were consuming Naswar for at least 1 year. Subjects with history of tobacco usage other than Naswar were excluded from the test group.

Additionally, subjects suffering from diabetes mellitus, obesity, cardiovascular, pulmonary or renal disorders or taking any antioxidant vitamin supplements were also excluded from the study. Ethical approval was obtained from the institutional ethical committee at the University of Karachi.

To assess the impact of duration of Naswar usage, Naswar users were further subdivided into three groups: Group 1: Subjects using Naswar for a duration of <5 years (*n* = 15), Group 2: Subjects using Naswar for duration of 6–10 years (*n* = 13) and Group 3: Subjects using Naswar for a duration of >10 years (*n* = 21). A questionnaire was filled to obtain information on age, occupational history, duration and frequency of Naswar usage.

#### Samples

Ten milliliter of venous blood was drawn from the antecubital vein under aseptic conditions after 12 h fast in vacutainers containing no additives. The blood was allowed to clot and serum separated within 30 min after centrifuging at 3000 rpm for 10 min and aliquots were stored at −20 °C.

### Laboratory measurement

IL-6 and IL-1ß were quantified by enzyme-linked immunosorbent assay (ELISA) (ABCAM catalogue number ab46027 and ab46052 respectively). The lower limits of detection of the kits (supplier's data) were as follows: IL-1β, 6.5 pg · mL-1; IL-6, 2 pg · mL-1; Samples with concentrations of IL-1β below the limit of detection were assigned a cytokine concentration value that was one half of the lower limit of detection rather than a zero value. IL-6 concentrations were above the lower limit of detection in all samples. FT3, FT4 and TSH were determined by enzyme-linked immunosorbent assay (ELISA) (Biocheck catalogue number BC-1006, BC-1008, BC1001 respectively). According to kits’ manufacturer, normal reference range for FT3 is 1.4–4.2 pg/ml, for FT4 0.8–2.0 ng/dl and for TSH in adults 21–54 years is 0.4–4.2μU/ml.

### Statistical analysis

The data was analyzed by statistical programme (SPSS) using student's independent samples *t*-test. One way Anova followed by post hoc Tukey test was applied to assess parameters in Naswar users grouped according to duration of Naswar usage. Pearson’s correlation coefficient was applied to assess correlations between parameters. *P* <0.05 was considered as statistically significant.

## Results

### Effect of Naswar usage on proinflammatory interleukins and thyroid function

IL-1β was found to be significantly lowered in Naswar users compared to the control group (*p* < 0.0001). On the other hand, no difference in the levels of serum IL-6 between Naswar users and the control group were found. Additionally, both FT3 and FT4 were significantly raised in Naswar users compared to the control group (*p* < 0.001) whereas no significant difference in the levels of TSH between Naswar users and the control group was found (Table 1).

**Table 1:** Effect of Naswar on proinflammatory interleukins and thyroid function

	Controls	Naswar Users
	*N* = 31	*N* = 49
IL-1β (pg/ml)	24.19 ± 0.73	16.27 ± 1.22 **
IL-6 (pg/ml)	7.69 ± 0.70	10.62 ± 1.17 ^NS^
FT4 (ng/dl)	1.79 ± 0.09	3.63 ± 0.06*
FT3 (pg/ml)	2.20 ± 0.15	4.76 ± 0.37*
TSH (μU/mL)	2.05 ± 0.21	1.60 ± 0.15 ^NS^

Experimental details are given in Methods section. Values are mean ± SEM. Statistical analysis was performed using student’s *t*-test. The significance of the difference is indicated by *p < 0.001 and **p < 0.0001 from controls

*NS* non-significant, *IL* interleukin, *FT3* free triiodohyronine, *FT4* free thyroxine, *TSH* thyroid stimulating hormone

### Effects of Naswar usage on pro inflammatory interleukins and thyroid function in Naswar users grouped according to duration of its usage

Serum IL-1β levels were found to be significantly lowered in all the three groups of Naswar users compared to the control group whereas no significant difference between the levels of IL-6 in all the three groups of Naswar users versus the control group was depicted. Both FT3 and FT4 were consistently raised in all the three groups of Naswar users as compared to controls. No significant difference in serum TSH was found in any of the three groups as compared to control (Table 2).

**Table 2:** Pro inflammatory interleukins and thyroid function in Naswar users grouped according to duration of its usage

	Controls	Naswar users grouped according to duration of Naswar usage		
		<5 years	6–10 years	>10 years
N	31	15	13	21
Interleukin 1β (pg/ml)	24.19 ± 0.73	13.46 ± 2.16*	15.29 ± 3.19*	17.62 ± 1.63*
Interleukin 6 (pg/ml)	7.69 ± 0.70	12.93 ± 3.24	7.03 ± 0.77	10.45 ± 1.32 ^NS^
FT3 (pg/ml)	2.20 ± 0.15	5.34 ± 0.65**	4.31 ± 1.0	4.81 ± 0.49**
FT4 (ng/dl)	1.79 ± 0.09	3.42 ± 0.07**	3.91 ± 0.18	3.66 ± 0.08**
TSH (μU/mL)	2.05 ± 0.21	1.52 ± 0.17 ^NS^	1.36 ± 0.24 ^NS^	1.81 ± 0.31 ^NS^

Experimental details are given in Methods section. Values are mean ± SEM. Statistical analysis was performed using one-way ANOVA followed by post hoc Tukey’s test. The significance of the difference is indicated by *p < 0.05 and **p < 0.01 from controls

*NS* non-significant, *FT3* free triiodohyronine, *FT4* free thyroxine, *TSH* thyroid stimulating hormone

### Correlations between IL-1β and IL-6 versus FT3, FT4 and TSH in Naswar users

**Fig. 1:**
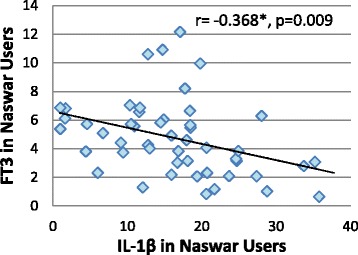
Correlation between IL-1β versus FT3 in Naswar users. FT3 = free triiodothyronine, IL = Interleukin. *Correlation is significant at the 0.01 level

FT3 was found to be significantly inversely related to serum IL-1β in Naswar users (Fig. 1) whereas no other significant correlations were depicted.

## Discussion

In the present study, various parameters related to the endocrinological and inflammatory responses in Naswar users were evaluated.

IL-1β was found to be significantly decreased in Naswar users compared to the controls whereas no difference in the levels of IL-6 between Naswar users and controls was found. These findings might be attributed to the immuno suppressant effects of nicotine present in Naswar as nicotine has been shown to exert an inhibitory effect on the release of the pro inflammatory cytokines IL-1β and IL-6 [25] as is also reflected by persistently decreased levels of IL-1β in Naswar users regardless of duration of Naswar consumption.

This is the first study evaluating the serum levels of IL-1β and IL-6 in Naswar users. However, a study conducted on mice showed increased plasma levels of IL-6 and IL-1β following an intra cerebroventricular injection of low dose nicotine. However, the maximum doses of peripherally administered nicotine failed to produce such a response [26]. Another study conducted on alveolar macrophages of chronic smokers has shown a defect in the release but not production of IL-1 from the alveolar macrophages [27]. Further, a study on an aqueous extract of smokeless tobacco showed increased production of TNF α and IL-1β following low doses of the extract [28]. Additionally, another study showed consistently raised levels of IL-6, IL-1β and platelet derived growth factor in the alveolar cells that were stimulated by antigen present in tobacco leaves [29]. Also, an imbalance in the circulating levels of interleukins in tobacco smokers has been reported and studies have shown significantly increased levels of plasma IL-6 in smokers compared to non-smokers [7, 8, 30].

Conversely, a study conducted on cigarette smoke extract to evaluate the levels of cytokines showed inhibition of TNF alpha, IFN-gamma, IL-2 and IL-1β indicating the presence of inhibitors of cytokine production in the extract [14]. Various studies have also shown decreased levels of various pro inflammatory cytokines in gingival clevicular fluid of smokers [31, 32].

The present study showed increased levels of FT4 and FT3 concentrations as compared to controls, whereas no difference between the levels of TSH in controls and Naswar users was found. However, the levels of TSH in Naswar users were on the lower limit of the normal range. We found no study depicting the levels of thyroid hormones in Naswar or any other smokeless tobacco product. However, there are studies evaluating thyroid hormone levels in cigarette smokers who have been reported to suffer from lowered TSH levels and raised T3 and T4 levels as compared to non-smokers [33]. Similar findings have been reported in passive smokers also [20]. Another study reported higher free T4 and free T3 levels as well as low TSH levels in smokers compared to non-smokers [34]. Low TSH levels in smokers have been reported in various other studies also [18, 35]. Additionally, Balhara and Deb reviewed the effects of tobacco on thyroid function and concluded that almost all functions of the thyroid gland are affected by tobacco smoking [36]. Thus, it could be inferred that nicotine exerts a stimulatory effect on the release of thyroid hormones thereby suppressing the release of TSH [19]. On the contrary, various studies have shown no relation between the levels of serum TSH and smoking [37–39].

Although, cytokines can also be produced by thyroid gland, they also target it resulting in disease [40] and increased levels of cytokines such as IL-6 and IL-1β have been associated with lowered T3 and T4 levels in non-thyroidal illnesses [41]. IL-1β down regulates the synthesis of thyroglobulin [42] and Thyroid peroxidase [43] and also causes inhibition of iodide organification [44] thereby leading to decreased thyroid hormone levels [45, 46]. Correlation between FT3, FT4 and TSH versus interleukins was determined in the present study in order to evaluate any association between Hypothalamic Pituitary Thyroid axis and interleukins in relation to Naswar consumption. A significant inverse correlation between FT3 and IL-1β in Naswar users was found that might reflect the effect of nicotine on thyroid hormones and interleukins. No association found between FT3, FT4, TSH versus IL-6 might be due to the normal levels of latter.

## Conclusion

Lowered levels of interleukin 1β in Naswar users indicate immunosuppressant effects of nicotine present in Naswar. In addition to this, raised levels of free T3 and free T4 in Naswar users, which although were in the upper limit of normal, depict the altered effects of Naswar usage on thyroid function.

## Abbreviations

FT3, free triiodothyronine; FT4, free thyroxine; IL, interleukin; TSH, thyroid stimulating hormone
